# Two‐stage portal flow modulation for volume‐augmented grafts in living donor liver transplantation: Rat model validation

**DOI:** 10.1002/ame2.70121

**Published:** 2026-01-07

**Authors:** Yuqi Gong, Yutong Chen, Zhoucheng Wang, Libin Dong, Zhengxing Lian, Kai Wang, Xiao Xu

**Affiliations:** ^1^ The Fourth School of Clinical Medicine Zhejiang Chinese Medical University Hangzhou China; ^2^ Zhejiang University School of Medicine Hangzhou China; ^3^ Key Laboratory of Integrated Oncology and Intelligent Medicine of Zhejiang Province Hangzhou First People's Hospital Hangzhou China; ^4^ General Surgery, Cancer Center, Department of Hepatobiliary & Pancreatic Surgery and Minimally Invasive Surgery, Zhejiang Provincial People's Hospital (Affiliated People's Hospital) Hangzhou Medical College Hangzhou China; ^5^ Hepatobiliary Center The First Affiliated Hospital of Nanjing Medical University Nanjing China; ^6^ Institute of Organ Transplantation Zhejiang University School of Medicine Hangzhou China

**Keywords:** animal models, living donor liver transplantation, liver regeneration, portal vein

## Abstract

Graft procurement in adult living donor liver transplantation (LDLT) faces persistent challenges in balancing volumetric adequacy and donor safety. This study introduces two‐stage portal vein ligation and reperfusion for graft procurement in LDLT (PVLR‐LT), which aims to expand the left lateral lobe for achieving adequate grafts, thereby circumventing technical and anatomical limitations of conventional approaches. In a rat model, the PVLR‐LT group underwent selective portal vein ligation (step I) to induce targeted hypertrophy, followed by reperfusion and transplantation (step II). Outcomes were compared among PVLR‐LT, negative controls, and standard‐volume controls. Staged portal flow modulation effectively redistributed hepatic mass allocation, yielding grafts with graft recipient weight ratio approximately double that of negative controls and equivalent to standard‐volume controls. Donors experienced no mortality, with only transient enzyme elevation. Recipient survival in the PVLR‐LT group significantly exceeded that of the negative control group and was non‐inferior to that of the standard‐volume control group, while hepatic enzyme peaks were markedly lower than those in standard‐volume control recipients. This study provides a promising proof of concept, establishing the feasibility of using PVLR‐LT to convert the surgically straightforward left lateral segment into right lobe‐sized grafts through staged portal flow modulation and demonstrating the translational potential for laparoscopic LDLT.

## Graphical abstract

This study presents a two‐stage portal flow modulation strategy for graft procurement in living donor liver transplantation with the aim of expanding left lateral lobe grafts, thereby circumventing technical and anatomical limitations of conventional approaches. In a rat model, this approach achieved significantly higher recipient survival than that achieved with conventional same‐lobe‐graft transplants, and caused less hepatic injury than standard same‐volume‐graft transplants.

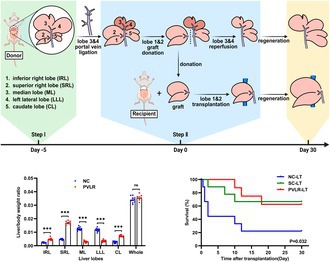

## 1 INTRODUCTION

In the face of a significant shortage of deceased liver donors, living donor liver transplantation (LDLT) remains crucial for end‐stage liver disease. However, LDLT inherently carries the risk of small‐for‐size syndrome, a severe complication characterized by insufficient graft volume leading to early dysfunction.^1^ Current standards recommend maintaining the graft volume to standard liver volume ratio at ≥40% and the graft recipient weight ratio at ≥0.8% to reduce the risk of small‐for‐size syndrome.^2^, ^3^ Consequently, securing adequately sized grafts while ensuring donor safety has persistently represented the defining challenge in LDLT.

Among graft type options in LDLT, left lateral segment grafts are advantageous due to their superficial abdominal position and relatively straightforward vascular and biliary anatomy. These characteristics simplify donor surgery and enable laparoscopic techniques.^4^, ^5^ However, their small size often inadequately supports adult recipients. Right lobe grafts provide sufficient volume for recipients, yet pose challenges: (1) Laparoscopic right lobe harvesting poses substantially greater technical challenges than left lateral segment procurement^6^; (2) the controversial inclusion of the middle hepatic vein in right lobe grafts creates decision‐making dilemmas requiring meticulous donor‐recipient risk balancing.^7^, ^8^, ^9^ Hence, developing a living donor hepatectomy technique capable of reconciling the anatomical simplicity of left lateral resection with right lobe‐level graft volumes has emerged as a pivotal frontier for advancing adult laparoscopic LDLT.

The application of staged surgery to augment the volume of the targeted liver segments offers a promising solution to the critical issue of graft volume insufficiency. This principle is strongly supported by the clinical success of associating liver partition and portal vein ligation for staged hepatectomy (ALPPS), a procedure developed for oncological patients requiring rapid liver hypertrophy (40%–80% volume increase within 6–9 days) prior to major resections.^10^ Through strategic staged modulation of portal flow, ALPPS demonstrates that controlled hepatic regeneration can be achieved, as evidenced by its reliable production of adequate future liver remnants. These mechanistic insights establish a scientific foundation for adapting staged regenerative strategies to overcome size limitations in left lateral segment LDLT.

In this study, we introduce two‐stage portal vein ligation and reperfusion for graft procurement in living donor liver transplantation (PVLR‐LT), a novel procedure designed to redefine graft acquisition in LDLT. PVLR‐LT focuses on converting the surgically straightforward left lateral segment into right lobe‐sized grafts through staged portal flow modulation. Theoretically, this approach ensures graft volume adequacy and donor safety while circumventing the technical challenges of laparoscopic right lobe procurement and the surgical controversies associated with middle hepatic vein allocation. We conducted systematic preclinical studies in rodents to validate its technical feasibility and safety.

## 2 MATERIALS AND METHODS

### 2.1 Animals

All experimental procedures involving animals adhered to the 3R principles and complied with the Animals in Research: Reporting In Vivo Experiments (ARRIVE) guidelines. These protocols were reviewed and approved by the Institutional Animal Care and Use Committee at Zhejiang Center of Laboratory Animals (approval number: 20010515). Male Sprague–Dawley rats were procured from Zhejiang Center of Laboratory Animals (Hangzhou, China). All rats were housed in a controlled environment at a constant temperature of 23 °C, with a 12 h light/12 h dark cycle, and had free access to food and water.

### 2.2 PVLR‐LT procedure in the rat model

**FIGURE 1:**
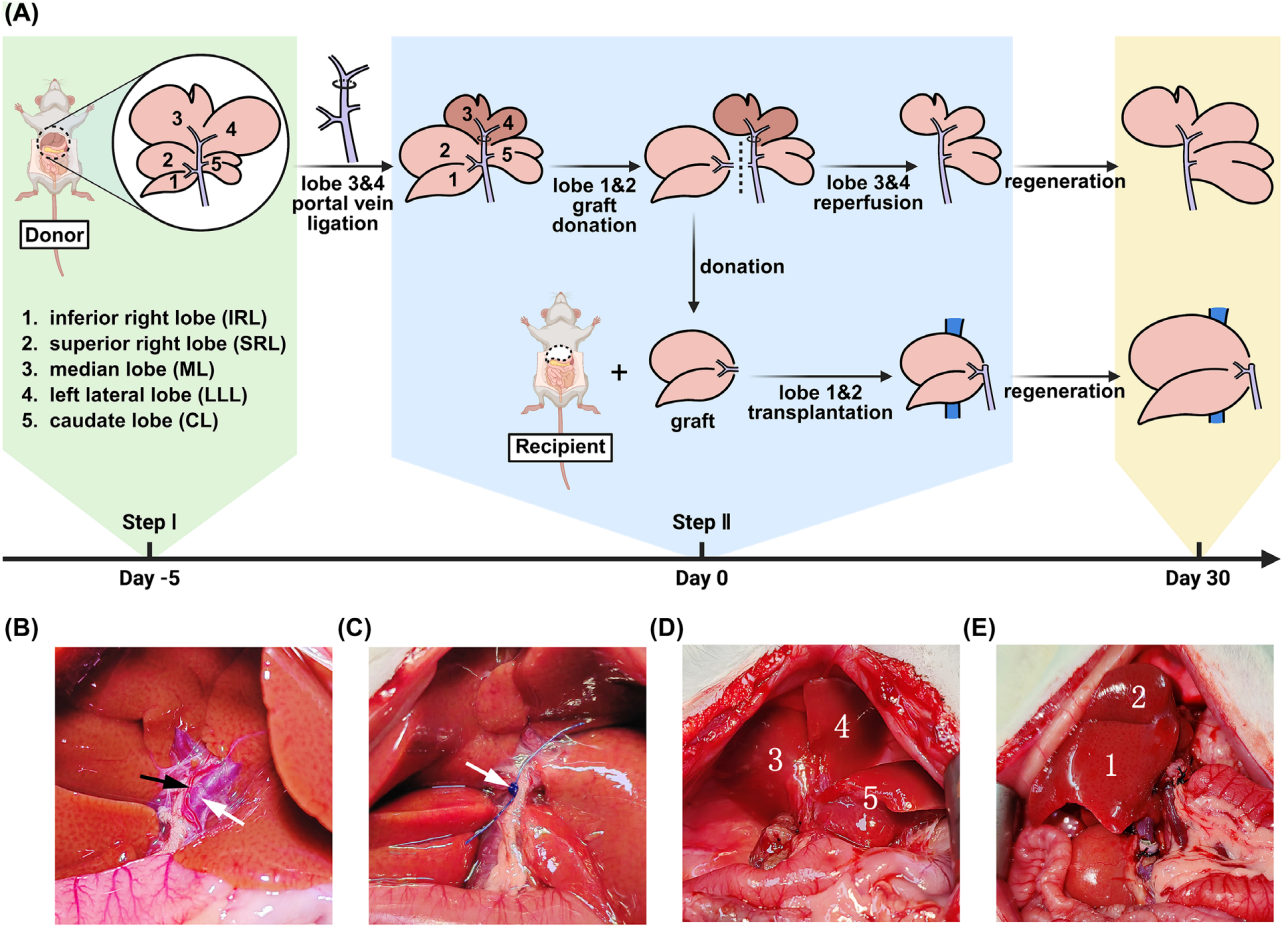
The fundamental procedure of the PVLR‐LT technique. (A) The PVLR‐LT procedure comprised two steps: Selective portal vein ligation of ML and LLL to induce hypertrophy in other lobes (step I), followed by reperfusion and transplantation of IRL and SRL grafts 5 days later (step II). (B) The Glisson system of ML and LLL shares a common trunk. The black arrow indicates branches of the hepatic artery, and the white arrow indicates branches of the portal vein. (C) Blood flow in the portal vein branch was occluded using 4–0 Prolene suture (white arrow). (D) On Day 0, the donor rat donated the graft, leaving the ML (3), LLL (4), and CL (5). (E) On Day 0, the recipient rat received the hypertrophied IRL (1) and SRL (2) grafts. CL, caudate lobe; IRL, inferior right lobe; LLL, left lateral lobe; ML, median lobe; SRL, superior right lobe.

Figure 1A illustrates the fundamental procedure for the PVLR‐LT technique. The rat liver is composed of the following five lobes: the inferior right lobe (IRL), the superior right lobe (SRL), the median lobe (ML), the left lateral lobe (LLL), and the caudate lobe (CL).^11^ In this study, IRL and SRL were chosen as the graft. Together, these two lobes make up around 20% of the liver's total volume, which is comparable to the human left lateral segment.

### 2.3 Step I – portal vein ligation (donor surgery)

On day −5, a midline laparotomy was performed on the donor rat. The first hepatic hilum was meticulously dissected to expose the portal venous branches supplying ML and LLL (Figure 1B). Selective ligation of these portal vein branches was achieved using 4–0 Prolene sutures, with careful preservation of corresponding hepatic arterial and biliary structures (Figure 1C).

### 2.4 Step IIa – reperfusion and graft harvesting (donor surgery)

Following the 5‐day ligation period, ML and LLL demonstrated a reduction in volume, while other hepatic lobes exhibited enlargement. On day 0, the previously placed portal vein ligatures were removed to restore perfusion to ML and LLL. Subsequently, IRL and SRL were resected as composite grafts, while preserving the remaining hepatic parenchyma (Figure 1D). Excised grafts were immediately submerged in the normal saline (0–4°C) for hypothermic preservation.

### 2.5 Step IIb – graft implantation (recipient surgery)

The recipient underwent midline laparotomy followed by ligation of the left phrenic vein, right adrenal vein, and hepatic artery. The common bile duct was then selectively transected. Vascular control was established through occlusion of the portal vein, infrahepatic inferior vena cava (IVC), and suprahepatic IVC. Total hepatectomy was performed, followed by orthotopic implantation of the donor graft into the hepatic fossa (Figure 1E). Microsurgical anastomosis of the suprahepatic IVC was completed using 8–0 Prolene in a running suture pattern. Portal venous and infrahepatic IVC reconstruction was accomplished via the cuff technique. Vascular clamps were sequentially removed to restore hepatic perfusion. Biliary continuity was reestablished using an intraluminal stent. Hemostasis was verified prior to abdominal closure in two anatomical layers.

### 2.6 Definition of groups

Donor and recipient survival analyses were conducted in separate cohorts as common practice,^12^, ^13^ because a paired model ensuring simultaneous survival of both was technically challenging and unstable. The lobe‐specific vascular and biliary tracts are too fine for reliable anastomosis, and the hepatic venous outflow shared with other lobes is difficult to isolate for partial graft. The donor experiments consisted of two groups: a two‐stage portal vein ligation and reperfusion (PVLR) group and a negative control (NC) group. The recipient experiments consisted of three groups: a PVLR‐LT group, a negative control for liver transplantation (NC‐LT) group, and a standard‐volume control for liver transplantation (SC‐LT) group. Vascular reconstruction techniques in liver transplantation were standardized across PVLR‐LT/NC‐LT/SC‐LT groups, with graft composition being the sole variable.

- The PVLR group underwent the novel two‐stage living donor hepatectomy. This group was designed to evaluate the impact of this surgery on donors.
- The NC group received living donor hepatectomy, serving as a control for comparison with the PVLR group.
- The PVLR‐LT group received IRL and SRL grafts via the novel PVLR‐LT procedure to evaluate its impact on recipients.
- The NC‐LT group underwent standard partial liver transplantation with IRL and SRL (mimicking human left lateral lobe grafts) to serve as the same lobe graft control for conventional techniques.
- The SC‐LT group underwent standard partial liver transplantation with IRL, SRL, and LLL (volumetrically matched to PVLR‐LT and simulating human right lobe grafts) to serve as the same volume graft control for conventional techniques.

### 2.7 Statistical analysis

GraphPad Prism 8.0.0 (GraphPad Software) generated graphs and presented quantitative data as mean ± SEM. For statistical comparisons (IBM SPSS Statistics 25.0), Student's *t* test was used for two groups, one‐way ANOVA for multiple groups, and repeated‐measures ANOVA for longitudinal data. Survival was analyzed by the Log‐Rank (Mantel‐Cox) test. Statistical significance was defined as *p* < 0.05.

Additional methods details are provided in the Supporting Information.

## 3 RESULTS

### 3.1 Step I portal flow modulation induces hepatic redistribution in rats

Based on the rodent liver regeneration timeline spanning from peak proliferation (1–3 days) to substantial completion (7 days) post‐ligation,^14^, ^15^ we conducted a study to compare the effects of 5‐day and 7‐day intervals between the two stages. It revealed equivalent liver/body weight ratios but increased adhesions at 7 days (Figure S1). Accordingly, the 5‐day interval was selected for all subsequent experiments.

**FIGURE 2:**
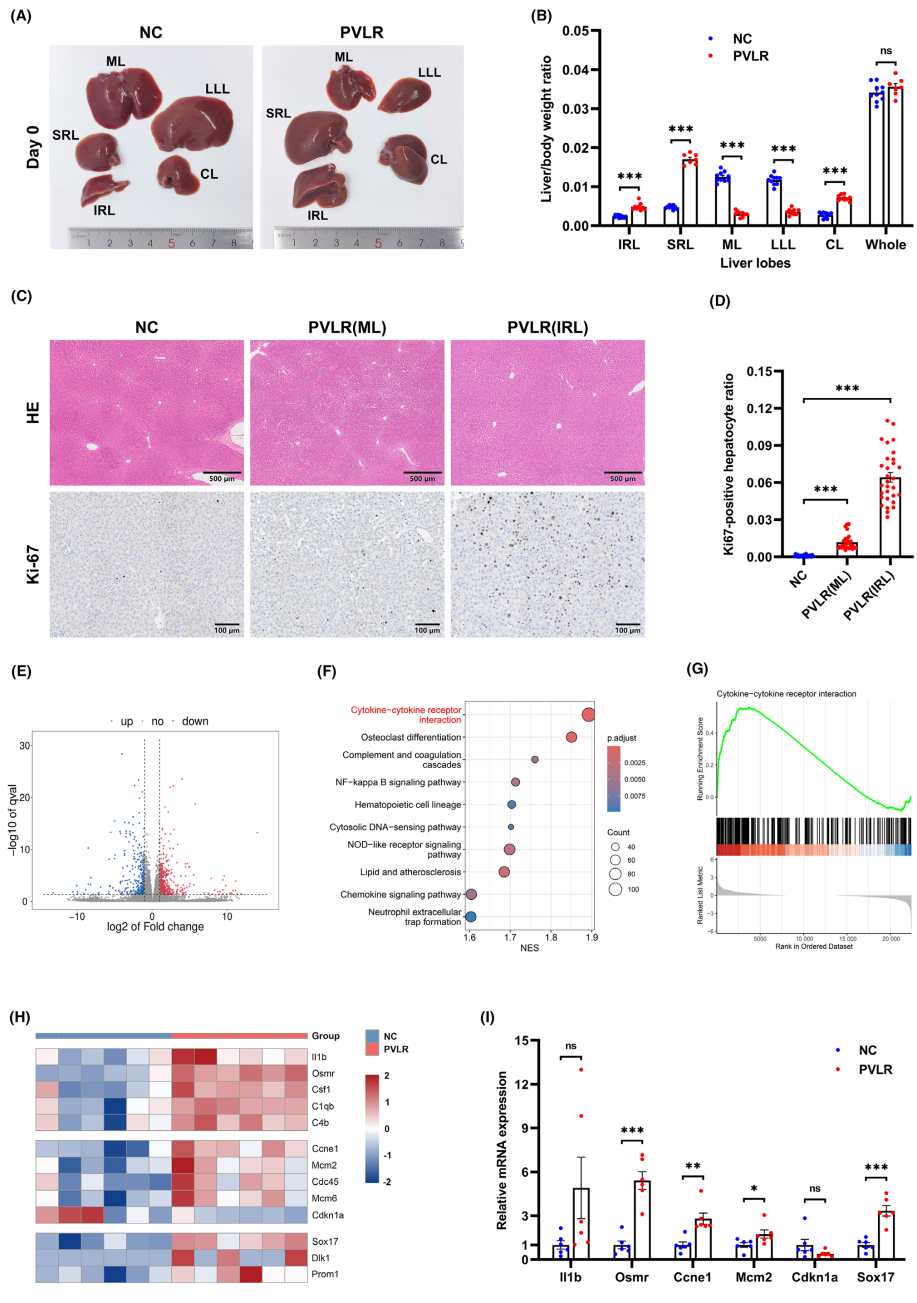
Portal flow modulation induces hepatic redistribution in rats. (A) Step I surgery altered the relative size of each liver lobe in 5 days. (B) The liver/body weight ratio of IRL, SRL, and CL was significantly higher in the PVLR group than in the NC group. ML and LLL were significantly smaller in the PVLR group compared to the NC group. The whole liver/body weight ratio showed no significant difference. (C, D) Liver tissues from IRL of NC group and PVLR group were collected for HE staining and immunohistochemical staining. (E) RNA sequencing of the IRL (PVLR vs. NC, *n* = 6/group) identified 1026 differentially expressed genes, with 700 genes upregulated and 326 downregulated in the PVLR group. (F) KEGG pathway analysis further identified predominant enrichment in cytokine signaling, NF‐κB activation, and complement system pathways. (G) GSEA plot showing the enrichment of the cytokine‐cytokine receptor interaction pathway. (H) Heatmap of differentially expressed genes related to regeneration initiation, cell cycle progression, and stemness activation. (I) qPCR validation of key regulators. mRNA levels of *Osmr*, *Ccne1*, *Mcm2*, and *Sox17* were significantly upregulated in the enlarged IRL of the PVLR group. IRL, inferior right lobe; SRL, superior right lobe; ML, median lobe; LLL, left lateral lobe; CL, caudate lobe; KEGG, Kyoto Encyclopedia of Genes and Genomes; NES, normalized enrichment score; GSEA, gene set enrichment analysis; Data were presented as mean ± SEM. Data in (B) and (I) were analyzed by unpaired two‐tailed Student's *t* test. Data in (D) were analyzed by one‐way ANOVA with Tukey's post hoc test. ns, not significant; *p < 0.05; **p < 0.01; ***p < 0.001.

We observed that 5‐day portal vein ligation induced hepatic mass redistribution. The liver weight‐to‐body weight ratios of portal‐perfused lobes (IRL, SRL, and CL) showed significant increases, whereas ligated segments (ML and LLL) exhibited size reduction (Figure 2A,B). Histological staining confirmed preserved hepatic lobular architecture with no evident inflammatory cell infiltration, fibrosis, or necrosis in all liver lobes. Immunohistochemical analysis demonstrated a significant elevation in Ki‐67‐positive hepatocyte ratios in hypertrophied lobes (Figure 2C,D). These findings demonstrate that the step I surgery safely achieves effective hypertrophy in portal‐perfused lobes.

### 3.2 Transcriptomic profiling uncovers a molecular program of regenerative initiation, proliferative execution and stemness activation

RNA sequencing of enlarged liver tissue identified 1026 differentially expressed genes after PVLR (Figure 2E). Kyoto Encyclopedia of Genes and Genomes (KEGG) pathway analysis revealed predominant activation of cytokine‐cytokine receptor interaction, complement system, and NF‐κB pathways (Figures 2F,G and S2A,B). Gene Ontology (GO) analysis also highlighted enrichment in biological processes related to regeneration, such as prolactin signaling (Figure S2C,D).

The heatmap displays differentially expressed genes from these pathways with established roles in regeneration initiation (Figure 2H). Guided by our hepatic enlargement phenotype, we identified distinct sets of differentially expressed genes associated with cell cycle control and stemness. The qPCR validation confirmed the significant upregulation of *Osmr*, *Ccne1*, *Mcm2*, and *Sox17* (Figure 2I).

### 3.3 Portal hemodynamic changes of donor rats after step I surgery

**FIGURE 3:**
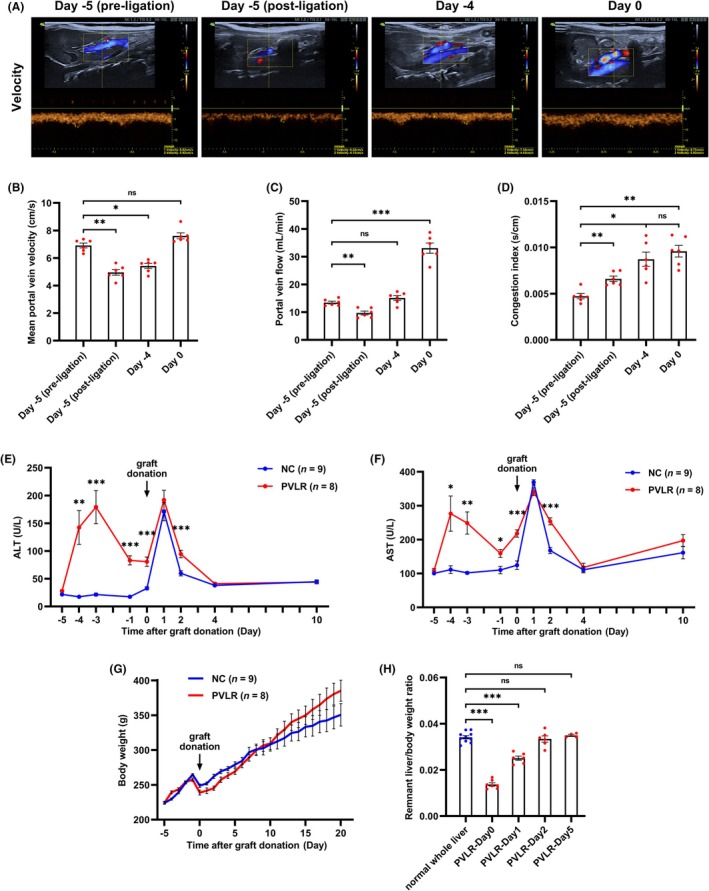
Safety profile of donor rats undergoing the PVLR‐LT protocol. PVLR group donors received selective portal vein ligation on day −5, followed by staged partial hepatectomy on day 0. In contrast, NC group donors received single‐stage hepatectomy exclusively on day 0. (A) Representative ultrasonography images showing the portal vein velocity in donor rats at the indicated time points. (B) Quantitative analysis of mean portal vein velocity. (C) Portal vein flow in donor rats after step I surgery. (D) Congestion index of portal vein in donor rats after step I surgery. (E) Serum ALT levels of donor rats in both groups. (F) Serum AST levels of donor rats in both groups. (G) Body weight changes of rats in both groups. (H) The remnant liver weight‐to‐body weight ratio in the PVLR group after step II surgery. ALT, alanine aminotransferase; AST, aspartate aminotransferase; Data were presented as mean ± SEM. Data in (B–D) were analyzed by two‐way repeated‐measures ANOVA. Data in (E) and (F) were analyzed by unpaired two‐tailed Student's *t* test. Data in (H) were analyzed by one‐way ANOVA with Tukey's post hoc test. ns, not significant; *p < 0.05; **p < 0.01; ***p < 0.001.

Serial ultrasonography was performed to monitor portal hemodynamics in donor rats following step I surgery (Figure 3A). The mean portal vein velocity decreased immediately after portal vein ligation but recovered to pre‐operative levels by day 0 (Figure 3B). Meanwhile, the portal vein diameter increased significantly from day −5 to day 0 (Figure S3A,B). The portal vein flow showed a transient reduction followed by a significant increase, markedly exceeding the pre‐operative baseline at day 0 (Figure 3C). The congestion index increased after ligation and remained stable after day −4 (Figure 3D).

### 3.4 PVLR‐LT ensures the safety of donor rats, with stable physiological recovery and rapid liver regeneration

To evaluate donor safety in the PVLR‐LT procedure, PVLR group donors received selective portal vein ligation on day −5, followed by staged partial hepatectomy on day 0. We observed transient elevation of ALT in donor rats after both step I and step II surgeries. However, peak levels showed no significant difference compared to the NC group (single‐stage hepatectomy performed solely on day 0) (Figure 3E). A similar trend was observed for AST levels (Figure 3F). No mortality occurred, and the body weight in both groups showed an upward trend throughout the experiment (Figure 3G). By postoperative day 2 following step II surgery, the remnant liver weight‐to‐body weight ratio in the PVLR group had returned to baseline whole‐liver levels (Figure 3H). These results collectively demonstrate great tolerance of the novel protocol by donor rats.

### 3.5 PVLR‐LT recipients exhibit survival benefits with favorable recovery dynamics

**FIGURE 4:**
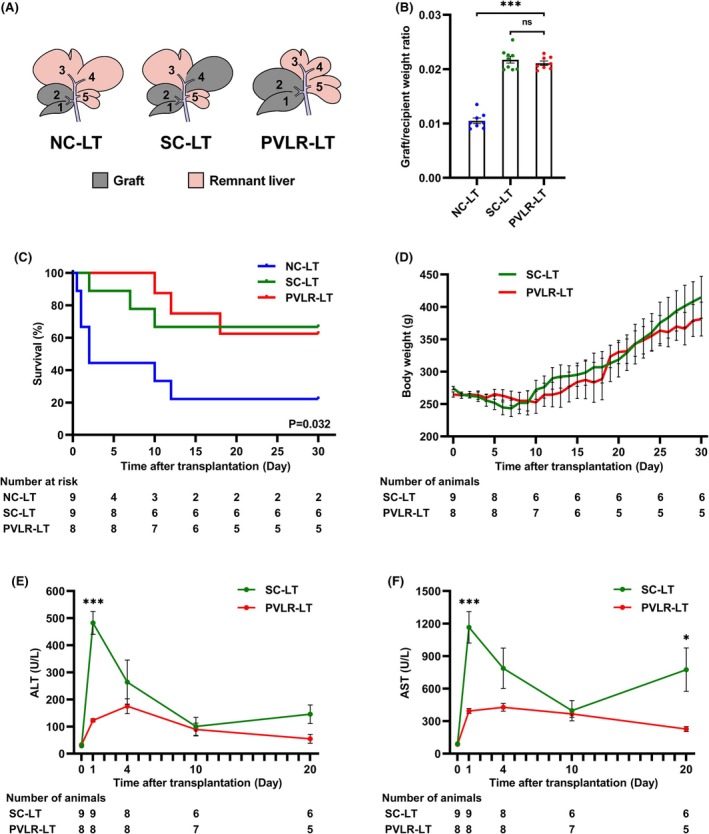
Recipient outcomes following PVLR‐LT in rats. (A) PVLR‐LT group recipients received IRL and SRL grafts through the novel PVLR‐LT procedure, while two control groups utilized conventional techniques: The NC‐LT group underwent standard partial liver transplantation, with IRL and SRL grafts serving as the same‐lobe control, and the SC‐LT group received IRL, SRL, and LLL grafts to establish the same‐volume graft control. (B) The graft/recipient weight ratios of the PVLR‐LT, NC‐LT, and SC‐LT groups on day 0. (C) PVLR‐LT recipients achieved a 30‐day survival rate of 62.5%, significantly higher than the NC‐LT survival rate of 22.2% (*p* = 0.034) and statistically non‐inferior to the 66.7% survival rate in the SC‐LT group (*p* = 0.950). (D) Body weight changes of recipients after step II surgery. (E) Serum ALT levels of recipient rats in the PVLR‐LT group and SC‐LT group. (F) Serum AST levels of recipient rats in the PVLR‐LT and SC‐LT groups. The low survival rate in the NC‐LT group precluded meaningful analysis of body weight and hepatic enzyme data. IRL, inferior right lobe; SRL, superior right lobe; LLL, left lateral lobe; ALT, alanine aminotransferase; AST, aspartate aminotransferase; Data were presented as mean ± SEM. Data in (B) were analyzed by one‐way ANOVA with Tukey's post hoc test. Survival in (C) was compared by the Log‐rank (Mantel‐Cox) test, and *p* values for pairwise comparisons were adjusted by the Bonferroni method. Data in (E) and (F) were analyzed by unpaired two‐tailed Student's *t* test. ns, not significant; *p < 0.05; ***p < 0.001.

Recipient rats underwent partial liver transplantation on day 0 using grafts from the donors that had received selective portal vein ligation on day −5. Two control groups—same lobe graft control (NC‐LT group) and same volume graft control (SC‐LT group)—were established without preoperative interventions in donors (Figure 4A). The PVLR‐LT procedure produced grafts with graft recipient weight ratios approximately double that of NC‐LT grafts and equivalent to SC‐LT grafts (Figure 4B). Survival analysis revealed that PVLR‐LT recipients achieved a 30‐day survival rate of 62.5%, significantly higher than the NC‐LT survival rate of 22.2% and statistically non‐inferior to the SC‐LT survival rate of 66.7% (Figure 4C). After an initial 10‐day postoperative weight loss phase, both groups exhibited sustained weight recovery (Figure 4D). Hepatic enzyme profiling revealed that ALT and AST levels peaked within postoperative days 0–4 and gradually normalized, with PVLR‐LT recipients exhibiting significantly lower peaks than SC‐LT recipients (Figure 4E,F).

In addition, we implemented the protocol using isolated SRL grafts to assess the feasibility of PVLR‐LT for ultra‐low‐volume grafts. Although all recipient rats died within 15 days, PVLR‐LT significantly extended survival duration compared to the same‐lobe graft control group (Figure S4A–D).

## 4 DISCUSSION

The present study introduces a novel two‐stage surgical protocol termed PVLR‐LT. Animal experiments demonstrated that the step I procedure of PVLR‐LT could change the relative weight proportions among the different liver lobes in rats. Specifically, the lobes with preserved portal blood flow significantly enlarged, while the lobes with ligated portal veins reduced in size. We observed transient elevation of liver enzymes in donor rats after step I surgery. The hemodynamic data provide an explanation for this transient change. Portal branch ligation initially induced a brief period of elevated flow resistance. This phase quickly transitioned into a stable state of high‐volume portal flow, driven by progressive vessel dilation and the restoration of flow velocity, which supported the subsequent regenerative response. Following the step II surgery, the subsequent overlap in enzyme levels between PVLR and NC donors indicates a normal course of recovery after graft donation.

Among recipients, the PVLR‐LT group achieved survival parity with standard grafts in the SC‐LT group and significantly outperformed the NC‐LT group. However, the 30‐day survival rate of 62.5% in this rat model cannot be directly compared to outcomes in human LDLT, as the surgical techniques and perioperative management in this rodent model were significantly different and simplified when compared to clinical practice, including: (1) the absence of hepatic artery reconstruction; (2) the use of materials with inferior biocompatibility; (3) less perioperative management. Importantly, despite equivalent graft volumes, PVLR‐LT recipients exhibited significantly attenuated peak hepatic enzyme levels compared to SC‐LT recipients. This protective effect can be attributed to the hemodynamic preconditioning of the graft during step I. By first encountering a period of elevated resistance and then adapting to a high‐flow environment, the graft was effectively primed to tolerate the portal hyperperfusion that follows transplantation. The decline and convergence of enzyme levels in both groups in the days after transplantation demonstrate that the preconditioned grafts successfully navigated the high‐risk period of initial reperfusion injury. Moreover, the experiment with isolated SRL grafts confirmed that, despite eventual mortality, PVLR‐LT could extend survival even with ultra‐low‐volume grafts. Nevertheless, this outcome also underscores that the technique's success remains contingent upon careful pre‐procedural assessment to ensure an adequate baseline graft volume.

The regenerative mechanism underlying PVLR‐LT shares similarities with portal vein ligation, portal vein embolization, and ALPPS. Ligation of portal branches to targeted liver lobes alters hemodynamics in the remnant lobes. Based on established evidence, this hemodynamic shift likely promotes regeneration through shear stress, hypoxia, and hyperperfusion.^16^, ^17^, ^18^ Notably, ligated lobes with preserved hepatic arterial perfusion avoid ischemic necrosis, enabling their re‐expansion upon subsequent reperfusion. Our transcriptomic data, validated by qPCR, delineate a coordinated molecular response associated with PVLR‐induced liver regeneration. The significant upregulation of *Osmr* aligns with established evidence that Oncostatin M signaling through its receptor is a potent activator of hepatocyte proliferation via the JAK–STAT3 pathway.^19^ This occurred alongside transcriptional changes in other key regulators: an upward trend in cytokine Il1b, the significant induction of cell cycle drivers *Ccne1* and *Mcm2*, and a downward trend in cycle inhibitor Cdkn1a. These concerted shifts in gene expression are indicative of pro‐proliferative state.^20^, ^21^ Furthermore, the significant upregulation of *Sox17*, a key marker of multipotent stem/progenitor cells, indicates activation of a regenerative reservoir beyond the self‐replication of mature hepatocytes.^22^ These findings position the PVLR procedure as an effective stimulus that coordinately engages key proliferative and stemness‐related programs, providing a molecular rationale for its capacity to augment graft volume.

A key translational consideration is the hepatic anatomical disparity between species. Unlike the rat liver with its separate lobes and minimal vascular connections, the human liver's continuous parenchyma facilitates rapid development of collateral circulation after partial portal vein ligation. If applied to humans, portal vein ligation alone would therefore be insufficient, as collateral flow would dampen the regenerative stimulus and prolong the regeneration process.^23^ Thus, its clinical application would likely require combination with parenchymal transection, akin to the ALPPS procedure, to achieve effective graft isolation and volume augmentation. This necessary transection introduces the specific risk of bile leakage. However, this risk can be mitigated by employing refined techniques such as partial parenchymal transection to reduce the raw cut surface area and meticulous bipolar cauterization of minor bile ducts.^24^ Another consideration is the potential risk of excessive portal hypertension resulting from portal flow modulation. Managing this hemodynamic consequence necessitates precise preoperative assessment and diligent postoperative monitoring of portal pressure to ensure donor safety. Furthermore, the potential influences of interspecies differences in portal hemodynamics and liver regeneration kinetics warrant further investigation in more physiologically relevant large‐animal models. These strategies are essential to balance the risk against the substantial benefit of obtaining a volumized graft.

PVLR‐LT is ultimately intended for human LDLT as a means of volumetrically expanding the left lateral lobe to achieve sufficient grafts. This approach carries several advantages: (1) The surgical procedure for left lateral lobe resection is technically simpler than resection of other lobes; (2) There is no issue of middle hepatic vein allocation, ensuring unobstructed venous drainage of all liver parts; (3) The size of the graft can be controlled flexibly by adjusting the timing of portal vein ligation.

In human application, the PVLR‐LT procedure could be implemented using minimally invasive approaches. Step I surgery shares technical similarities with ALPPS, for which laparoscopic or robot‐assisted techniques are well documented.^25^, ^26^ Interventional embolization is a viable alternative to surgical ligation, minimizing hilar dissection and subsequent adhesion formation.^27^ The use of biodegradable embolic microspheres even enables clinicians to precisely regulate the timing of portal vein branch embolization.^28^ For step II, laparoscopic living donor liver resection has been widely performed. Pure laparoscopic left lateral lobe living donor hepatectomy has even been listed as a standard practice by the 2019 International Laparoscopic Liver Society (ILLS) and the Asian‐Pacific Hepato‐Pancreato‐Biliary Association (A‐PHPBA) consensus.^5^

## 5 CONCLUSION

In summary, this study provides a proof of concept in a rodent model for the PVLR‐LT method, demonstrating its potential to enhance LDLT by volumetrically expanding the left lateral segment through a two‐stage surgical approach to acquire sufficient grafts. To bridge the translational gap between preclinical findings and clinical implementation, we plan to conduct further validation through porcine and non‐human primate studies. These investigations will facilitate the establishment of PVLR‐LT as a mature and reliable technology in the field of LDLT.

## AUTHOR CONTRIBUTIONS

**Yuqi Gong:** Conceptualization; data curation; formal analysis; investigation; methodology; visualization; writing – original draft. **Yutong Chen:** Data curation; formal analysis; investigation; methodology; visualization; writing – original draft. **Zhoucheng Wang:** Conceptualization; supervision; writing – review and editing. **Libin Dong:** Formal analysis; visualization. **Zhengxing Lian:** Methodology. **Kai Wang:** Conceptualization; funding acquisition; supervision; writing – review and editing. **Xiao Xu:** Funding acquisition; project administration; supervision; writing – review and editing.

## FUNDING INFORMATION

This study was supported by grants from the General Program of National Natural Science Foundation of China (No. 82470683); National Key Research and Development Program of China (No. 2021YFA1100500); The Innovation Team of Hangzhou Medical College (No. CXLJ202401); Key Research & Development Program of Zhejiang Province (No. 2022C03108); Ningbo Top Medical and Health Research Program (No. 2024020818).

## CONFLICT OF INTEREST STATEMENT

The authors declare no conflicts of interest.

## ETHICS STATEMENT

All experimental procedures involving animals were reviewed and approved by the Institutional Animal Care and Use Committee at Zhejiang Center of Laboratory Animals (approval number: 20010515).

## PERMISSION TO REPRODUCE MATERIAL FROM OTHER SOURCES

The manuscript contains no material reproduced from other sources.

## Supporting information


Figure S1.

Figure S2.

Figure S3.

Figure S4.

Caption S1:

Appendix S1:

## Data Availability

All data are available from the corresponding author upon reasonable request.
