# Supplementary figures and images for: Two‐stage portal flow modulation for volume‐augmented grafts in living donor liver transplantation: Rat model validation
Source: Animal Model Exp Med. 2026 Jan 7;8(12):2288–97. doi: 10.1002/ame2.70121 (PMC13020041; doi:10.1002/ame2.70121)

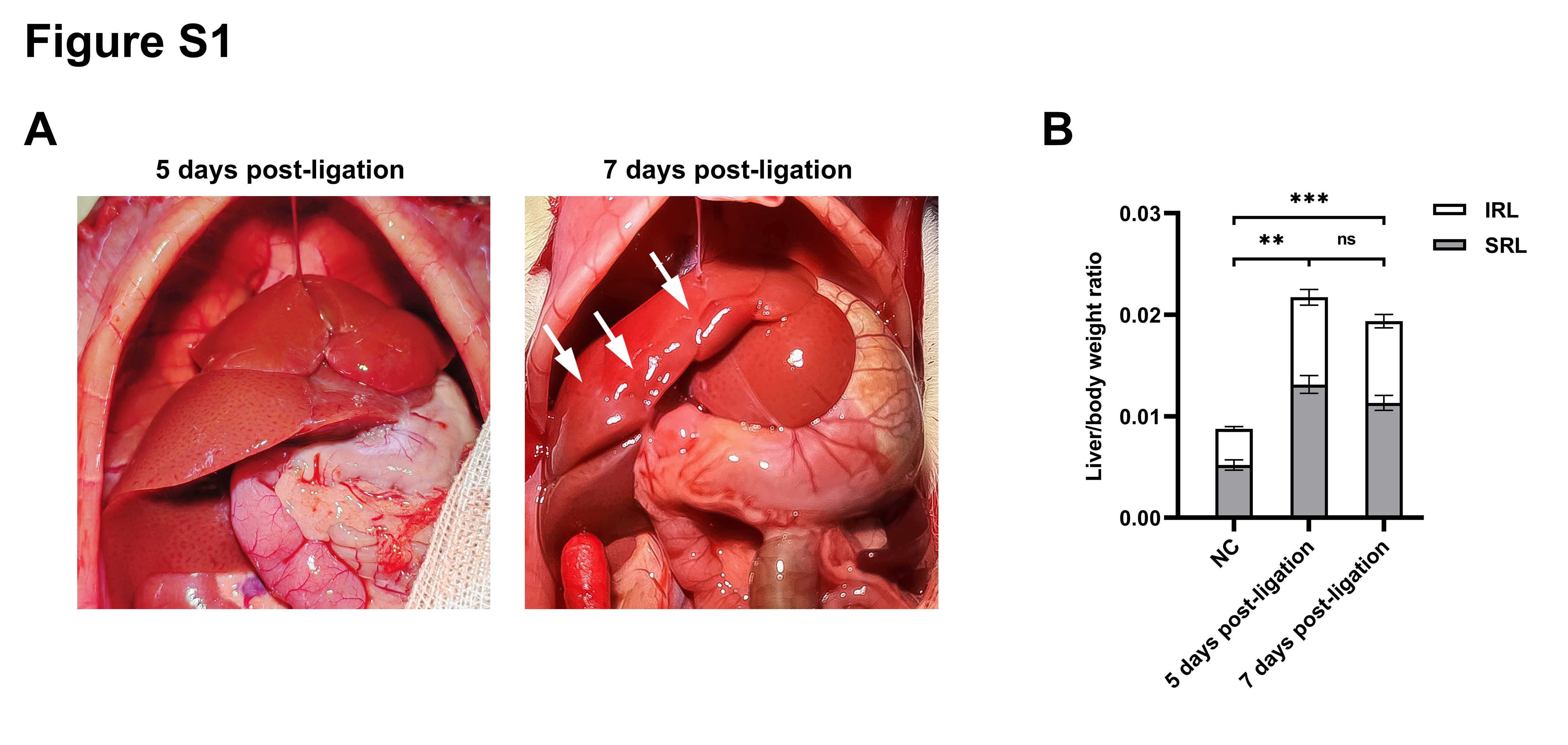

Supplement: Supplementary file 1 — Figure S1. [file AME2-8-2288-s005.tif]

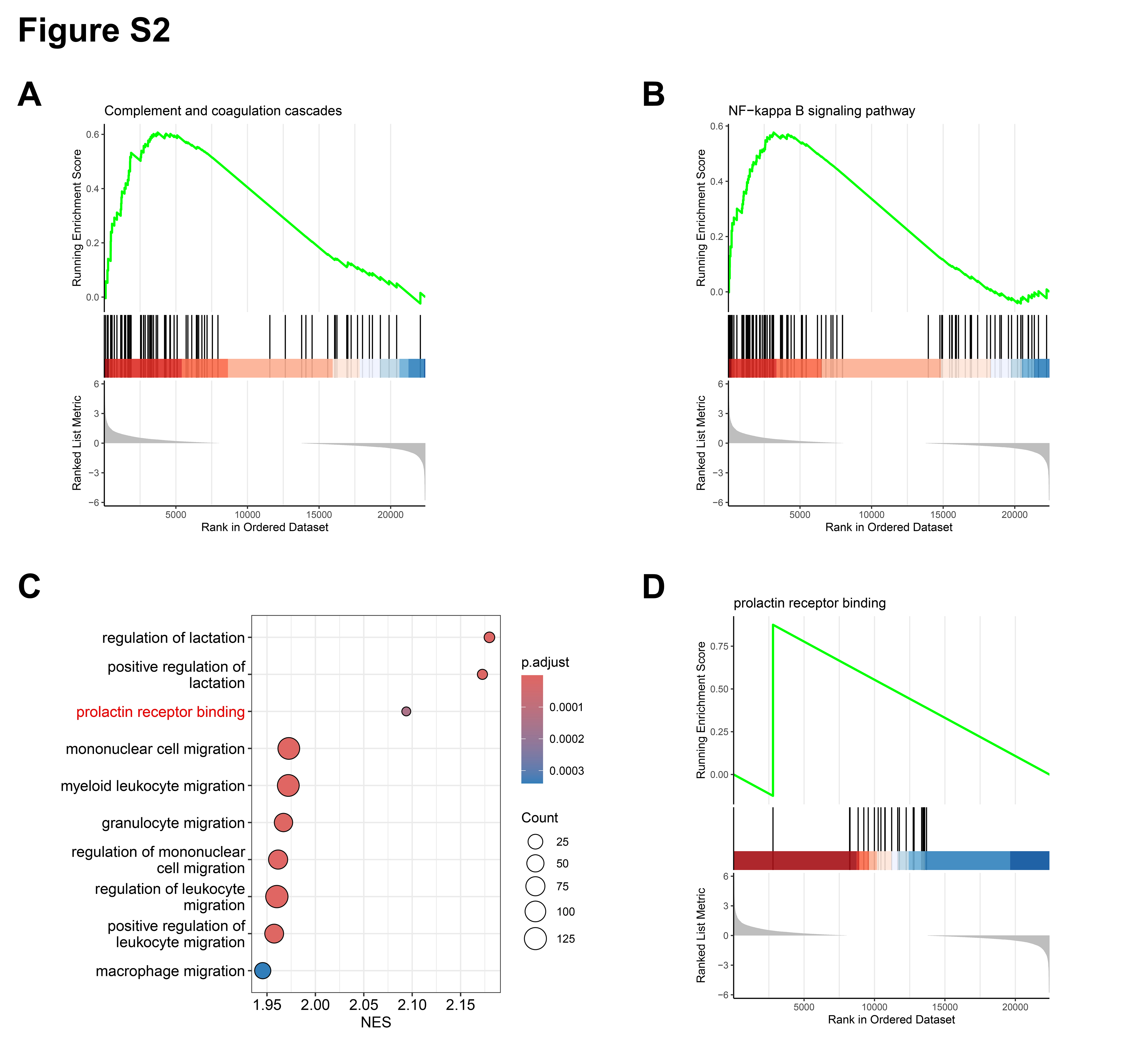

Supplement: Supplementary file 2 — Figure S2. [file AME2-8-2288-s003.tif]

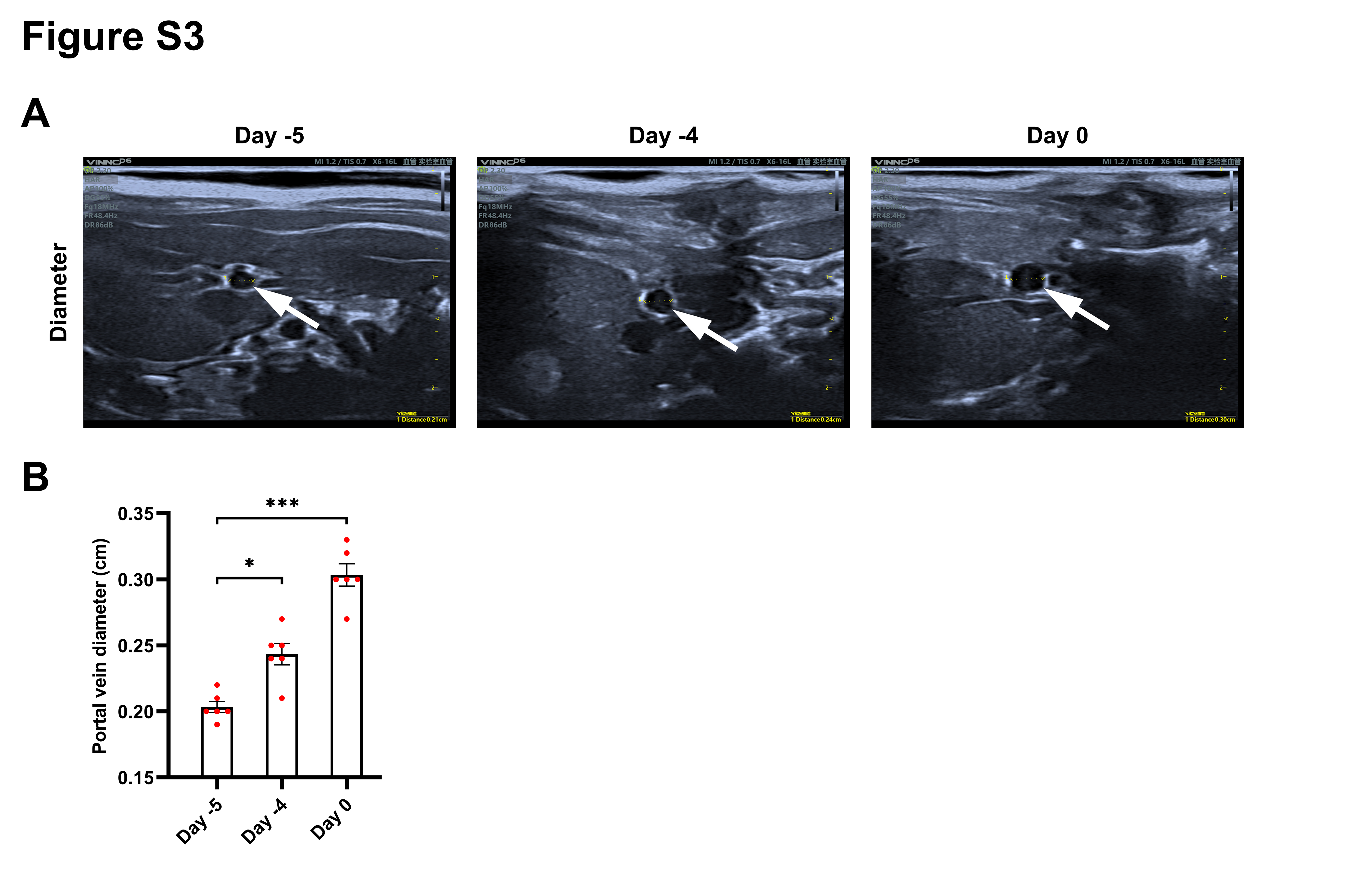

Supplement: Supplementary file 3 — Figure S3. [file AME2-8-2288-s004.tif]

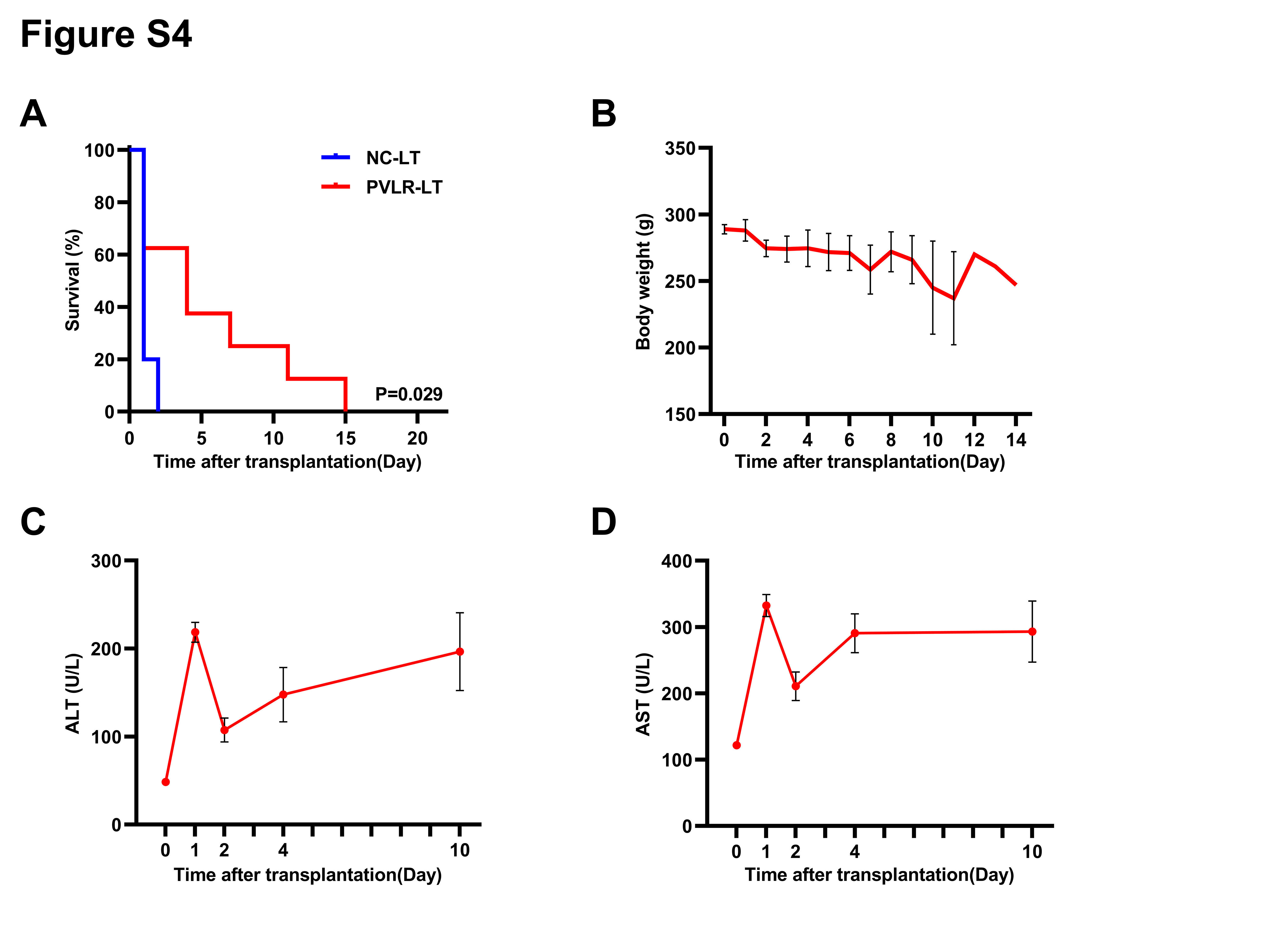

Supplement: Supplementary file 4 — Figure S4. [file AME2-8-2288-s001.tif]
