# Supplementary material for: Two‐stage portal flow modulation for volume‐augmented grafts in living donor liver transplantation: Rat model validation
Source: Animal Model Exp Med. 2026 Jan 7;8(12):2288–97. doi: 10.1002/ame2.70121 (PMC13020041; doi:10.1002/ame2.70121)
Supplement: Supplementary file 5 — Caption S1: [file AME2-8-2288-s006.docx]

**SUPPLEMENTARY FIGURE LEGENDS**

**Figure S1. Determination of the Optimal Interval for the Two-Stage PVLR-LT Procedure.​**

**(A)** Representative images of liver adhesion formation (white arrow) at 5-day versus 7-day intervals after step I portal vein ligation. **(B)** The liver/body weight ratio of IRL and SRL in the NC group, 5-day, and 7-day interval groups.

IRL, the inferior right lobe; SRL, the superior right lobe; Data were presented as mean ± SEM. Data in **(B)** were analyzed by one-way ANOVA with Tukey’s post hoc test. ns, not significant; **P<0.01; ***P<0.001.

**Figure S2. Transcriptomic Enrichment Profiles of Regeneration-Associated Pathways in Portal Flow-Redistributed Liver Lobes.**

**(A)** GSEA plot showing the enrichment of the complement and coagulation cascades pathway. **(B)** GSEA plot illustrating the enrichment of the NF-κB signaling pathway. **(C)** GO analysis of significantly enriched terms revealed biological processes broadly linked to prolactin signaling and leukocyte migration regulation; **(D)** GSEA plot showing the enrichment of the prolactin receptor binding pathway.

GSEA, gene set enrichment analysis; GO, Gene Ontology.

**Figure S3. Serial Assessment of Portal Vein Diameter in Donor Rats after Step I Surgery.​**

**(A)**​​ Representative transverse-view ultrasonography images showing the portal vein (white arrow) for diameter measurement. **(B)** Quantitative analysis of portal vein diameter in donor rats after step I surgery. Data were presented as mean ± SEM. Data in **(B)** were analyzed by two-way repeated-measures ANOVA. *P<0.05; ***P<0.001.

**Figure S4. Assessment of ultra-low volume grafts in PVLR-LT.**

PVLR-LT group (n=8) recipients received enlarged SRL grafts through the novel PVLR-LT procedure, while NC-LT group (n=5) underwent standard partial liver transplantation with SRL grafts. The survival rate **(A)**, body weight **(B)**, serum ALT **(C)**, and serum AST **(D)** are presented. Insufficient survival rates in the NC-LT group precluded meaningful analysis of body weight and hepatic enzyme data.

ALT, alanine aminotransferase; AST, aspartate aminotransferase. Data were presented as mean ± SEM. Survival in **(A)** was compared by the Log-rank (Mantel-Cox) test, and P-values for pairwise comparisons were adjusted by the Bonferroni method.
