# Supplementary material for: Two‐stage portal flow modulation for volume‐augmented grafts in living donor liver transplantation: Rat model validation
Source: Animal Model Exp Med. 2026 Jan 7;8(12):2288–97. doi: 10.1002/ame2.70121 (PMC13020041; doi:10.1002/ame2.70121)
Supplement: Supplementary file 6 — Appendix S1: [file AME2-8-2288-s002.docx]

**SUPPLEMENTARY METHODS**

**Assessment of Portal Hemodynamics**

Portal hemodynamics were evaluated under isoflurane anesthesia in donor rats using VINNO D6 ultrasound system (VINNO Corporation, China). Portal vein diameter was measured with the probe held perpendicular to the vessel. Mean portal vein velocity was determined using pulsed-wave doppler with the probe aligned parallel to the vessel, and was calculated as the average of the maximum and minimum velocities.

Portal vein flow (ml/min) = π × [diameter (cm)/2]^2^ × mean velocity (cm/s) × 60.

Congestion index (s/cm) = π × [diameter (cm)/2]^2^ / mean velocity (cm/s).

**Histological and Immunohistochemical Staining**

Liver tissues from IRL were fixed, paraffin-embedded, sectioned, and processed for hematoxylin-eosin (H&E) staining. For Ki-67 assessment, dewaxed and rehydrated sections underwent antigen retrieval, followed by sequential blocking and incubation with rabbit anti-Ki-67 antibody (Abcam, UK) and horseradish peroxidase-conjugated secondary antibody. Diaminobenzidine staining and hematoxylin counterstaining were performed. Ki-67 positive hepatocyte ratio was calculated as the ratio of diaminobenzidine-stained nuclei to total nuclei (≥1,000 nuclei/field) across ten randomly selected 100× fields per section.

**RNA Sequencing and Bioinformatics Analysis**

The IRL of the liver was collected from rats in the PVLR and NC groups at day 0 as tissue samples. RNA libraries were constructed and sequenced on the illumina NovaseqTM 6000 platform (LC-Bio Technology Co., Ltd, China). Differential gene expression analysis was performed using DESeq2, with thresholds set at |log₂(fold change)| ≥1 and adjusted p-value <0.05. Gene Ontology (GO) and Kyoto Encyclopedia of Genes and Genomes (KEGG) pathway enrichment analyses were performed using hypergeometric tests, with significance thresholds set at a Benjamini-Hochberg adjusted p-value (p. adjust) < 0.05.

**Liver Biochemical Analysis**

Under isoflurane anesthesia, blood was collected from rat orbits using capillary glass tubes. After clotting at room temperature for 30 minutes, samples were centrifuged (2000g, 15 minutes), and serum alanine aminotransferase (ALT) and aspartate aminotransferase (AST) levels were quantified using an automatic biochemical analyzer.
